# Individual influenza A virus mRNAs show differential dependence on cellular NXF1/TAP for their nuclear export

**DOI:** 10.1099/vir.0.018564-0

**Published:** 2010-05

**Authors:** Eliot K. C. Read, Paul Digard

**Affiliations:** Division of Virology, Department of Pathology, University of Cambridge, Tennis Court Road, Cambridge CB2 1QP, UK

## Abstract

The influenza A virus RNA-dependent RNA polymerase produces capped and polyadenylated mRNAs in the nucleus of infected cells that resemble mature cellular mRNAs, but are made by very different mechanisms. Furthermore, only two of the 10 viral protein-coding mRNAs are spliced: most are intronless, while two contain unremoved introns. The mechanism(s) by which any of these mRNAs are exported from the nucleus is uncertain. To probe the involvement of the primary cellular mRNA export pathway, we treated cells with siRNAs against NXF1, Aly or UAP56, or with the drug 5,6-dichloro-1-*β*-d-ribofuranosyl-benzimidazole (DRB), an inhibitor of RNA polymerase II phosphorylation previously shown to inhibit nuclear export of cellular mRNA as well as influenza virus segment 7 mRNAs. Depletion of NXF1 or DRB treatment had similar effects, inhibiting the nuclear export of several of the viral mRNAs. However, differing degrees of sensitivity were seen, depending on the particular segment examined. Intronless HA mRNA and spliced M2 or unspliced M1 transcripts (all encoding late proteins) showed a strong requirement for NXF1, while intronless early gene mRNAs, especially NP mRNA, showed the least dependency. Depletion of Aly had little effect on viral mRNA export, but reduction of UAP56 levels strongly inhibited trafficking and/or translation of the M1, M2 and NS1 mRNAs. Synthesis of NS2 from the spliced segment 8 transcript was, however, resistant. We conclude that influenza A virus co-opts the main cellular mRNA export pathway for a subset of its mRNAs, including most but not all late gene transcripts.

## INTRODUCTION

Influenza A virus has a negative-sense RNA genome split into eight viral (v) RNA segments. Unusually for an RNA virus without a DNA-encoding stage, vRNA synthesis occurs in the nucleus (Amorim & Digard, 2006). Here, the vRNAs are transcribed by the viral RNA-dependent RNA polymerase to produce 10 major species of capped and polyadenylated mRNA. Although these transcripts are structurally similar to mature host mRNAs, their terminal modifications are achieved through mechanistically distinct routes. Viral mRNAs contain 5′-cap structures that are synthesized by the cellular capping machinery but recycled through the process of ‘cap-snatching’, in which a cap-dependent endonuclease activity in the viral polymerase generates capped oligonucleotide primers from cellular pre-mRNAs (Neumann *et al.*, 2004). Poly(A) tails are synthesized by the viral polymerase stuttering on a poly(U) tract near the 5′-end of each vRNA segment (Neumann *et al.*, 2004), rather than by the host 3′-end processing machinery and poly(A) polymerase. The maturation pathway of most of the viral transcripts also differs from that of cellular mRNAs. While the majority of cellular pre-mRNAs contain introns that are completely removed before nuclear export, most influenza mRNAs (those from segments 1–6, encoding PB2, PB1, PA, HA, NP and NA, respectively, in their largest ORFs) are intronless. Furthermore, although segments 7 and 8 contain introns, the majority (∼90 %) of the primary transcripts are exported unspliced to produce the abundant M1 and NS1 polypeptides, respectively, while the processed minority encode the M2 and NS2/NEP proteins (Lamb & Horvath, 1991). Thus, the 10 protein-coding influenza virus mRNAs can be classified into three structural classes: intronless, intron-containing but unspliced and fully spliced. Viral mRNAs can also be subdivided into ‘early’ (segments 1–3, 5 and the unspliced segment 8 transcript) and ‘late’ (segments 4, 6, 7 and spliced segment 8) classes, based on the temporal expression patterns of their protein products (Hatada *et al.*, 1989; Inglis & Mahy, 1979; Lamb *et al.*, 1978; Skehel, 1972). However, these structural or kinetic classifications do not neatly correspond.

The mechanism(s) by which influenza virus mRNAs reach the cytoplasm is unclear. Cellular mRNAs are predominantly exported in the form of messenger ribonucleoprotein complexes (mRNPs) via the NXF1/TAP pathway. Formation and recruitment of these mRNPs to the NXF1 export pathway is closely coupled to Pol II transcription and pre-mRNA maturation, including capping, splicing and polyadenylation steps (Erkmann & Kutay, 2004; Fasken & Corbett, 2005; Howe, 2002). During synthesis and processing, a cellular mRNA dynamically acquires a complex set of proteins, including various heterogeneous nuclear ribonucleoproteins (hnRNPs), poly(A)-binding proteins and the transcription-export (TREX) complex. TREX, consisting of the THO complex, UAP56 (BAT1) and Aly (REF/THOC4), is primarily recruited to the pre-mRNA during splicing (Masuda *et al.*, 2005; Strasser *et al.*, 2002) but also through interactions with the 5′-cap structure (Cheng *et al.*, 2006; Nojima *et al.*, 2007). Aly then recruits NXF1 and its co-factor p15, which displaces Aly and allows NXF1/p15 to bind the RNA directly (Hautbergue *et al.*, 2008). NXF1–p15 interactions with the nuclear pore complex then direct export of the mRNP into the cytoplasm. The synthesis of influenza mRNAs by a viral polymerase and the fact that most are either intronless or contain residual introns raises the question of how, or even if, they are fed into the NXF1 pathway. Alternative cellular mRNA export pathways exist, the best characterized of which utilizes the CRM1/exportin 1 factor (Carmody & Wente, 2009; Hutten & Kehlenbach, 2007). Other nuclear replicating viruses such as herpesviruses manage to export intronless mRNAs by encoding adaptor proteins that functionally replace Aly and link the viral transcripts to the NXF1 pathway (Boyne *et al.*, 2008; Stamminger, 2008). Retroviruses export intron-containing mRNAs through *cis*-acting RNA sequences that (directly or via a viral adaptor protein) recruit either NXF1- or CRM1-dependent pathways (Cullen, 2003; Fontoura *et al.*, 2005). Indirect evidence argues both for and against influenza virus possessing analogous mechanisms. Viral gene expression is insensitive to leptomycin B (LMB) treatment (Elton *et al.*, 2001), as is the export of mRNAs from segments 6 and 7 (Amorim *et al.*, 2007; Wang *et al.*, 2008), arguing against the use of the CRM1 pathway. However, drugs that inhibit Pol II lead to reversible nuclear retention of segment 4 and 7 but not segment 5 mRNAs (Amorim *et al.*, 2007; Vogel *et al.*, 1994). Transcription-dependent export of cellular mRNA has also been demonstrated (Huang *et al.*, 1994; Tokunaga *et al.*, 2006), suggesting that at least some influenza mRNAs use cellular export machinery that is ordinarily coupled to Pol II transcription. Consistent with this, an siRNA screen for proteins involved in the early stages of influenza virus replication in *Drosophila* cells identified NXF1 as important for expression of a chimaeric reporter gene based on segment 6, as well as for overall virus replication in mammalian cells (Hao *et al.*, 2008). It has also been shown that influenza mRNAs from segments 2, 6 and 7 interact with NXF1 (Wang *et al.*, 2008). However, a direct test of whether NXF1 functions in the export of any or all the influenza virus mRNAs has not been reported. Furthermore, it has also been proposed that influenza virus NS1 antagonizes the export function of NXF1 as a means of contributing to host-cell shut off (Satterly *et al.*, 2007).

Accordingly, to clarify the mechanism(s) by which influenza A virus mRNAs are exported from the nucleus, we set out to directly test the involvement of the cellular NXF1 and CRM1 pathways. We found no evidence for the involvement of the latter system in influenza mRNA export, whereas export of all viral transcripts tested showed some degree of sensitivity to NXF1 levels. However, a gradient of dependence was evident, with late gene mRNAs being the most sensitive and the intronless early gene transcripts being the least sensitive.

## RESULTS

**Fig. 1.**
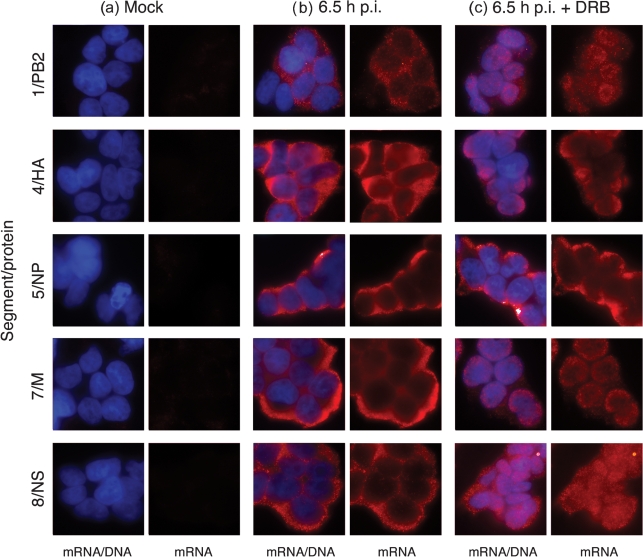
Effect of DRB on influenza virus mRNA localization. 293T cells were infected (or mock infected) with PR8 virus, treated where indicated with DRB at 90 min and 6.5 h p.i. localization of the indicated segments determined by FISH (red) and wide field fluorescence microscopy. Nuclei were counterstained with DAPI and false-coloured blue.

Previously, we showed that inhibition of RNA Pol II transcription blocked the nuclear export of segment 7 mRNAs (encoding M1/M2), but not segment 5 (NP) mRNA (Amorim *et al.*, 2007). Analysis of viral protein expression in the presence of the drug 5,6-dichloro-1-*β*-d-ribofuranosyl-benzimidazole (DRB), an inhibitor of processive Pol II elongation (Clement & Wilkinson, 2000; Fraser *et al.*, 1978; Tamm *et al.*, 1980), showed reductions in the synthesis of HA and NS1 which suggested that ongoing Pol II transcription was necessary for the export of other viral mRNAs than just that encoding M1 (Amorim *et al.*, 2007). However, the processing and export of cellular pre-mRNAs is also associated with the nuclear loading of protein factors that subsequently upregulate translation of the mature transcripts in the cytoplasm (Fasken & Corbett, 2005; Howe, 2002). We therefore tested directly whether DRB treatment inhibited export of other influenza virus mRNAs than those from segment 7. Cells were infected (or mock infected) with influenza A/PR/8/34 (PR8) virus and 90 min post-infection (p.i.) were either treated with DRB or left untreated. At 6.5 h p.i., the cells were fixed and analysed by fluorescence *in situ* hybridization (FISH) for the localization of positive-sense RNA from segments 1, 4, 5, 7 and 8. Only background levels of signal were seen from uninfected cells (Fig. 1a). In untreated cells all of the viral mRNAs tested were found to be predominantly cytoplasmic (Fig. 1b). When infected cells were treated with DRB, segment 5 mRNA still remained mostly cytoplasmic, while as expected (Amorim *et al.*, 2007) segment 7 transcripts showed pronounced nuclear retention (Fig. 1c). Segment 4 mRNA (encoding HA) also showed almost total nuclear retention after DRB treatment, while transcripts from segments 1 (PB2) and 8 (NS1/NS2) showed an intermediate phenotype with obvious nuclear retention, but with residual cytoplasmic staining still clearly visible (Fig. 1c). Thus, nuclear export of the majority of influenza mRNAs is sensitive to DRB treatment and the loss of NS1 and HA protein expression under these conditions (Amorim *et al.*, 2007; Tamm *et al.*, 1984) directly reflects reduced nuclear export of their mRNAs. However, a gradient of responsiveness to the inhibitor was apparent, with the transcripts of early genes generally showing reduced sensitivity than those of late genes.

**Fig. 2.**
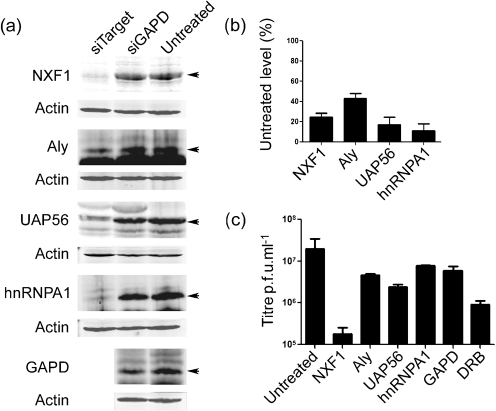
siRNA depletion of cellular mRNA export factors. (a, b) Lysates from infected siRNA-treated or mock-treated 293T cells at 6.5 h p.i. were analysed by SDS-PAGE and Western blotting for the indicated antigens. (b) Levels of the indicated polypeptides from replicate experiments were quantified and plotted as the mean±sem relative to untreated cells. (c) siRNA-treated cells were infected at an m.o.i. of 10 and output virus titres at 11 h p.i. from cells treated (or mock treated) with siRNAs targeting the indicated proteins determined by plaque assay. The mean±sd of three independent experiments is shown.

Given the requirement for ongoing Pol II transcription for both influenza and cellular mRNA export (Amorim *et al.*, 2007; Huang *et al.*, 1994; Tokunaga *et al.*, 2006), it was reasonable to hypothesize that the cellular mRNA export machinery was involved. Accordingly, we targeted key components of the cellular bulk mRNA export machinery: NXF1, Aly and UAP56, for siRNA-mediated knockdown. We also targeted hnRNPA1, a protein involved in cellular mRNP biogenesis, as well as glyceraldehyde-3-phosphate dehydrogenase (GAPD) as a control for non-specific siRNA effects. Both UAP56 and hnRNPA1 have been identified as interaction partners of influenza A virus NP/RNPs (Mayer *et al.*, 2007; Momose *et al.*, 2001). Lysates from siRNA-treated human embryonic kidney (293T) cells were first analysed by Western blotting to confirm successful knockdown of the target proteins. In all cases, a reduction in the levels of the targeted protein in comparison with control or untreated cells was apparent (Fig. 2a). When blots from replicate experiments were quantified, normalized to actin levels and compared with untreated cells, NXF1 levels were reduced by 75, Aly by 60, UAP56 by 80 and hnRNPA1 by 90 % at 48 h post-transfection (Fig. 2b), confirming successful reductions in expression.

To establish whether depletion of these cellular proteins affected influenza virus replication, siRNA-treated cells were infected with PR8 virus and the output titres determined by plaque assay. The effect of DRB on virus replication was also assessed, as a known inhibitor of influenza replication that functions at least in part by blocking viral mRNA export and/or gene expression (Amorim *et al.*, 2007; Tamm *et al.*, 1984); this treatment reduced virus titres by around 50-fold compared with untreated cells (Fig. 2c). Cells in which GAPD had been targeted for knockdown showed only a slight reduction in their ability to replicate virus, as did cells depleted of hnRNPA1 or Aly (Fig. 2c). However, targeting of NXF1 reduced the output virus titre approximately 100-fold, while knockdown of UAP56 reduced replication by nearly 10-fold (Fig. 2c). These data, consistent with a previous study that examined knockdown of NXF1 (Hao *et al.*, 2008), strongly suggested that NXF1 was involved in influenza replication, while UAP56 may also be important.

**Fig. 3.**
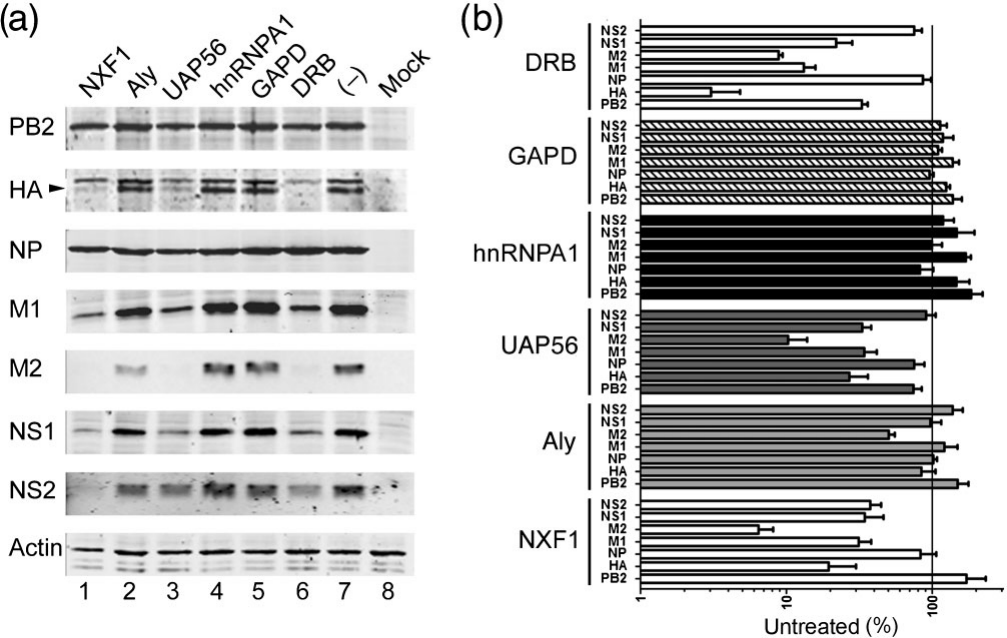
Effect of siRNA depletion of cellular mRNA export factors on viral gene expression (a, b) 293T cells were transfected with siRNAs against the indicated proteins [or mock transfected (–)] then infected or mock infected with virus. Cell lysates taken at 6.5 h p.i. were analysed by Western blotting for the indicated antigens. (b) Accumulation of the indicated viral polypeptides from replicate experiments were quantified and plotted as the mean±sem of the level seen in non-siRNA-treated infected cells after normalization for actin levels.

To better define the role of these cellular proteins in the influenza virus life cycle, we next examined viral protein expression in siRNA-treated cells. Lysates from cells treated with siRNAs (or later treated with DRB as a positive control) and then infected with virus were analysed by Western blotting for a panel of viral proteins, including the early class gene products PB2 (as a representative polymerase protein), NP and NS1, the late proteins HA and M1 and the two products of spliced mRNAs, M2 and NS2. RT-PCR analysis of the relative abundance of the M1 and M2 mRNAs indicated that none of the siRNA treatments had a major effect on splicing of segment 7 transcripts (data not shown). The levels of cellular actin were also examined to ensure equal quantities of cell lysate had been tested (Fig. 3a, bottom row). Viral proteins were readily detectable in samples from untreated infected cells, but not in mock infected cell lysates (Fig. 3a, lanes 7 and 8). As observed previously (Amorim *et al.*, 2007; Tamm *et al.*, 1984), DRB treatment profoundly decreased levels of the late viral proteins M1 and HA and reduced NS1 and M2 synthesis, while leaving NP and NS2 accumulation unaffected (Fig. 3a, lane 6). The partial retention of segment 1 mRNA seen after DRB treatment (Fig. 1) correlated with a threefold reduction in PB2 accumulation (Fig. 3a, lane 6; see Fig. 3b for quantification). When viral protein levels were examined in siRNA-treated cells, depletion of GAPD or hnRNPA1 had no effect (Fig. 3a, lanes 4 and 5; quantification in Fig. 3b). Depletion of Aly also had little consequence for viral protein levels, causing at the most a twofold reduction in M2 accumulation (Fig. 3a, lane 2). Knockdown of UAP56 had more effect; while PB2, NP and NS2 levels were unaffected, NS1, HA, M1 and M2 accumulation were all reduced by threefold or more, with M2 being the most severely affected (Fig. 3a, lane 3 and Fig. 3b). However, consistent with its effect on virus titre, depletion of NXF1 had the greatest effect on viral gene expression, severely reducing M2 and HA protein levels and also reducing M1, NS1 and NS2 accumulation by around threefold. Similarly to DRB treatment, NXF1 depletion had little effect on NP accumulation, while PB2 levels were also unaffected.

**Fig. 4.**
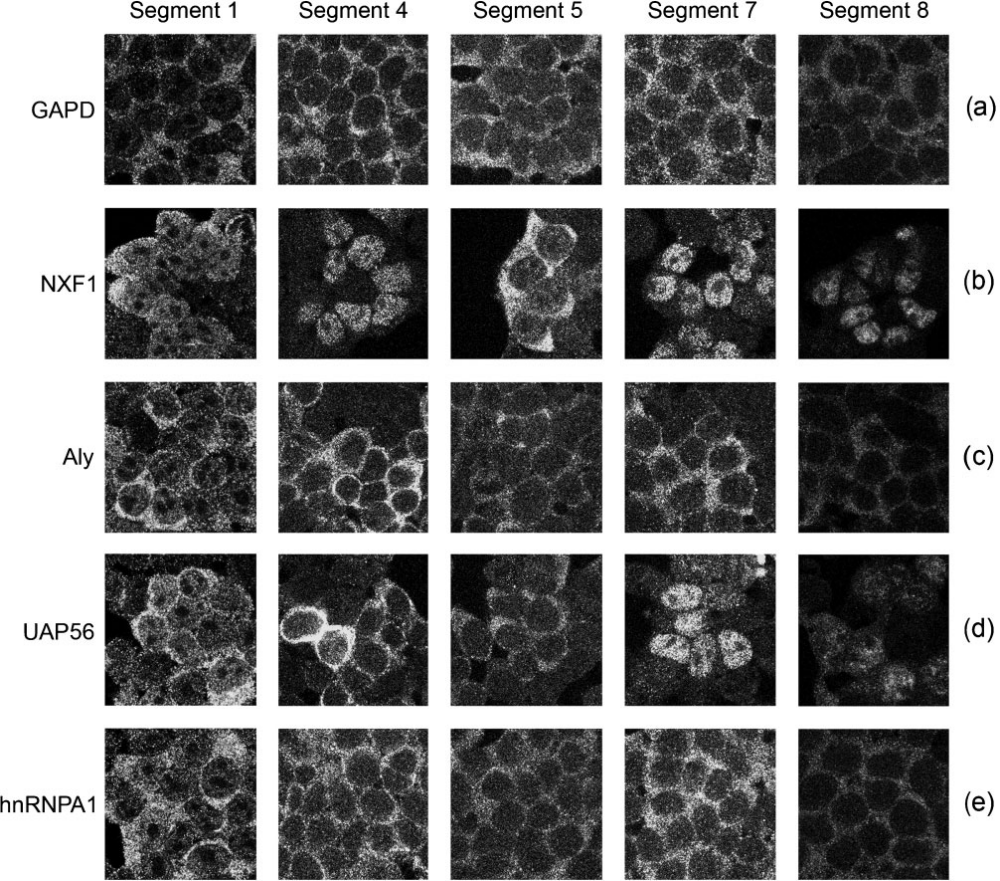
Effect of siRNA depletion of cellular mRNA export factors on viral mRNA localization. 293T cells were transfected with siRNAs against the labelled proteins, infected with virus and positive-sense transcripts from the indicated segments detected by FISH followed by confocal microscopy.

**Fig. 5.**
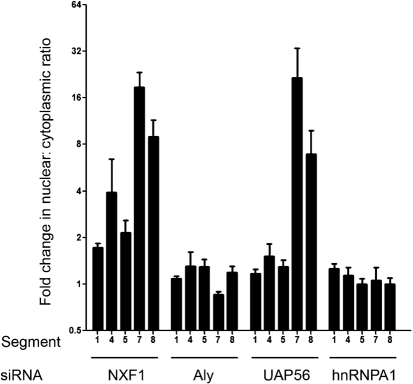
Quantitative analysis of the effect of siRNA depletion of cellular mRNA export factors on viral mRNA localization. The nuclear : cytoplasmic staining ratios of the FISH probes from replicate confocal FISH images of infected cells treated with siRNAs as described in Fig. 4 were determined and plotted as the fold change compared with the values seen in GAPD siRNA-treated cells. Error bars represent the sem.

Thus, reduced quantities of certain components of the cellular mRNA export machinery leads to much reduced synthesis of particular influenza virus proteins. The most obvious cause for this would be inhibition of viral mRNA export. To test this we infected siRNA-treated cells and analysed the localization of viral mRNA by FISH. Positive-sense transcripts from segments 1, 4, 5, 7 and 8 were all largely cytoplasmic in cells treated with siRNAs targeting GAPD (Fig. 4a), similar to the pattern seen in untreated cells (Fig. 1b). This pattern was not altered by siRNA depletion of either hnRNPA1 or Aly (Fig. 4e, c, respectively). Quantification of the nuclear : cytoplasmic ratio of staining intensity seen for the FISH probes from multiple cells confirmed that there was no substantial change in localization of any of the viral transcripts examined (Fig. 5). Depletion of UAP56 also had little effect on the localization of mRNAs from segments 1, 4 and 5, either by visual inspection (Fig. 4d) or by quantification of fluorescence intensity (Fig. 5). However, substantial nuclear retention was obvious for mRNAs from segments 7 and 8 (Fig. 4d), resulting in between 8 and nearly 30-fold increases in the relative amount of nuclear staining (Fig. 5). Depletion of NXF1 caused a similarly large decrease in the amount of cytoplasmic mRNA from segments 7 and 8, but pronounced nuclear retention of segment 4 was also apparent (Figs 4b and 5). A slight (around twofold) increase in nuclear : cytoplasmic staining ratios was also seen for segment 1 and 5 mRNAs. Thus, UAP56 is required for the export of segment 7 and 8 mRNAs, while NXF1 is required for the efficient nuclear export of most influenza A virus mRNAs, but to a degree that depends on the specific transcript examined. Also, the similar effects DRB treatment and NXF1 depletion have on viral mRNA localization coupled with previous work showing that DRB treatment inhibits bulk cellular mRNA export (Huang *et al.*, 1994; Tokunaga *et al.*, 2006) suggests that the drug inhibits NXF1-dependent mRNA export.

**Fig. 6.**
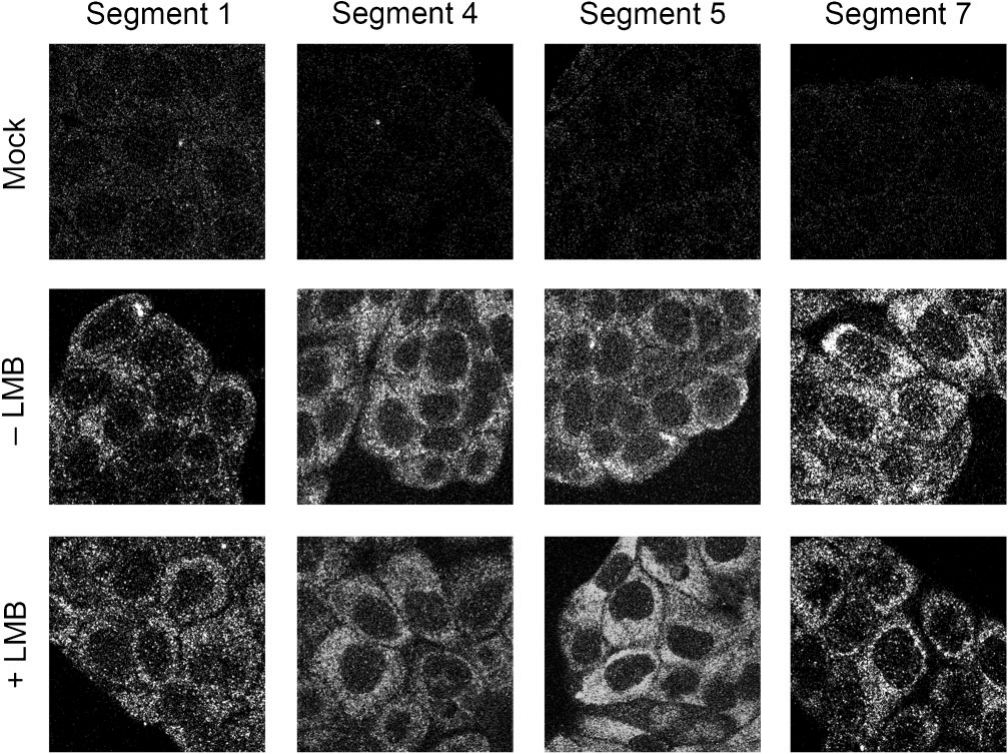
Effect of LMB treatment on viral mRNA localization. MDCK cells were infected or mock infected with virus, treated where indicated with LMB from 90 min p.i. and positive-sense RNA from specific segments (as labelled) detected by FISH and confocal microscopy at 6.5 h p.i.

The low sensitivity of intronless early gene transcripts to the effects of NXF1 depletion or DRB treatment could result from their use of a non-NXF1-dependent export pathway. Alternatively, these mRNAs might still use NXF1 but recruit it via a different, more efficient mechanism than that used by the late gene/spliced transcripts, which largely bypasses the blocks introduced by DRB or gene silencing. As a first test of these possibilities, we examined whether influenza virus mRNA export was sensitive to LMB, as CRM1 is the best delineated alternative pathway for mRNA nuclear export (Carmody & Wente, 2009; Hutten & Kehlenbach, 2007). Madin–Darby canine kidney (MDCK) cells were infected with virus, treated (or mock treated) with LMB from 1.5 h p.i. and viral mRNA localization examined by FISH. Parallel analysis of the intracellular localization of NP showed its LMB-dependent nuclear retention as expected (Elton *et al.*, 2001), confirming activity of the drug (data not shown). Again, positive-sense transcripts from segments 1, 4, 5 and 7 were largely detected in the cytoplasm in untreated cells (Fig. 6, middle panels). Also consistent with previous results (Amorim *et al.*, 2007; Wang *et al.*, 2008), localization of mRNA from segment 7 was unaltered by LMB treatment (Fig. 6, lower right). Moreover, trafficking of segment 1, 4 and 5 transcripts were also unaffected by the drug. Similar results were obtained in 293T and BHK cells (data not shown). Thus, while not eliminating the hypothesis that the early intronless viral mRNAs utilize a non-NXF1-dependent export pathway, these data indicate that they do not use a CRM1-dependent mechanism.

## DISCUSSION

**Fig. 7.**
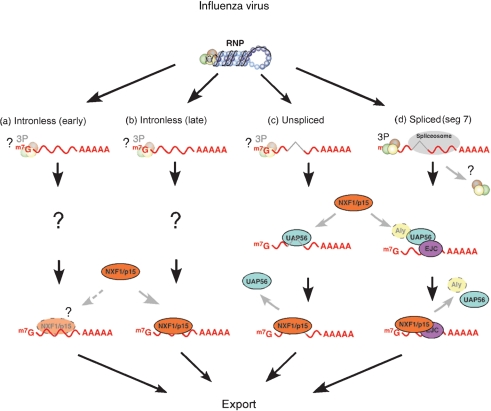
Cartoon model for influenza virus mRNA export. Viral mRNAs split into the four indicated conceptual classes are synthesized in the nucleus by vRNPs. (a) Intronless mRNAs encoding early gene products (PB2, NP, putatively PB1 and PA) show only weak dependence on NXF1 levels for nuclear export. If NXF1 is directly involved in their export, its means of recruitment are unknown. (b) Intronless mRNAs encoding late gene products (HA, probably NA) show strong dependence on NXF1 for export, but its means of recruitment is also uncertain. (c) Unspliced, intron containing mRNAs (M1 and NS1) show strong dependence on NXF1 and UAP56 levels for export. (d) Spliced segment 7 mRNA (M2) shows strong dependence on NXF1 and UAP56 levels and a weak dependence on Aly for its export, which is proposed to follow the normal cellular pattern of splicing dependent deposition of the exon junction complex followed by recruitment of UAP56, Aly and NXF1. The viral polymerase complex (3P) is also known to bind the 5′-end of this mRNA and may also interact with other viral mRNAs. NS2 mRNA is omitted from this model because of insufficient data.

Like any virus that transcribes its genome in the nucleus of infected cells, influenza A must ensure that its mRNAs reach the cytoplasm. Retroviruses and nuclear-replicating DNA viruses face a similar problem but for influenza, it is perhaps rendered more difficult by an RNA genome and consequent use of a virally encoded RNA-dependent RNA polymerase. We show here that despite not directly using Pol II to transcribe its genome, influenza virus nevertheless co-opts the cellular NXF1 export machinery for at least a subset of its mRNAs. We also show that not all viral mRNAs interact equally with this nuclear export pathway, with individual viral transcripts displaying a gradient of susceptibility to treatments that disrupt bulk mRNA export. Differing requirements for the helicase UAP56 were also seen. These data allow us to start building a model for how the individual viral mRNAs reach the cytoplasm (Fig. 7).

Our conclusion that the NXF1 pathway is used for influenza virus mRNA export is based on several strands of evidence. Firstly, it is supported by prior work showing that NXF1 is required for viral replication (Hao *et al.*, 2008) and that mRNAs from segments 2, 6 and 7 can be co-immunoprecipitated with NXF1 (Wang *et al.*, 2008). More directly, however, we show for the first time that loss of cellular NXF1 strongly inhibited nuclear export of segment 4, 7 and 8 mRNAs (Figs 4b and 5). The FISH probes we employed do not discriminate between spliced and unspliced transcripts from the latter two vRNAs, but synthesis of all four polypeptides encoded by the segments were reduced (Fig. 3), suggesting that export of the minority spliced species (especially that of M2) was also inhibited. Transport of segment 4, 7 and 8 mRNAs, as well as expression of the proteins they encode (excepting NS2), were also strongly retarded by the Pol II inhibitor, DRB (Figs 1 and 3). In contrast, the nucleo-cytoplasmic distribution of the intronless mRNAs from segments 1 and 5 were relatively unaffected by NXF1 depletion, displaying only a twofold change (Fig. 5) without a matching reduction in accumulation of the translation products (Fig. 3b). DRB also had little effect on NP mRNA localization or translation, while partial nuclear retention of segment 1 mRNA was seen, only a threefold drop in protein accumulation resulted (Figs 1 and 3b).

The selective nature of the defects in viral gene expression arising from NXF1 or UAP56 depletion also argue that the requirement for the cellular export machinery is direct (and not simply the consequence of lowered cell viability), since the siRNA-treated cells were still readily infectable with virus and able to support normal synthesis of certain viral polypeptides. This is also consistent with the findings of Hao *et al.* (2008) who found that NXF1 depletion of HEK cells did not dramatically affect cell viability over the time-spans used here.

The differing susceptibilities individual viral mRNAs showed to siRNA depletion of cellular export factors or DRB correlated better with the kinetic class of the viral gene product than with mRNA structure. Intronless transcripts for early gene products (in particular segment 5/NP mRNA) but also segment 1 (PB2) showed the least dependence on the NXF1 pathway (Fig. 7a), while late genes, including the intronless mRNA encoding HA, the spliced mRNA for M2 and the intron-containing but unspliced M1 message showing the clearest dependence (Fig. 7b–d). We have not examined the susceptibility of segment 6 (NA) mRNA to NXF1 depletion but Wang *et al.* (2008) showed an association between the two molecules, while Hao *et al.* (2008) reported that NXF1 depletion blocked expression of an artificial reporter mRNA based on segment 6. It therefore seems plausible that the NA mRNA has a similar export mechanism to the HA mRNA (Fig. 7b). The correlation between the degree of dependence on NXF1 and the kinetic class of the viral gene product is not perfect however, as expression of the late protein NS2 (from the spliced segment 8 mRNA) was less sensitive to DRB than expression of the early protein NS1 from the unspliced transcript (Fig. 3b) and the export of the majority population of positive-sense mRNA from segment 8 was inhibited by both DRB and NXF1 depletion (Figs 1, 4 and 5).

The question therefore arises of how the viral mRNAs are recruited to the NXF1/p15 pathway for export. Depletion of Aly, the most thoroughly characterized adaptor protein for cellular mRNA, had little effect on transport of viral messages (Figs 4 and 5) or protein expression (Fig. 3). This is perhaps surprising given the dependence cellular mRNAs show on Aly for export (Carmody & Wente, 2009; Cheng *et al.*, 2006; Nojima *et al.*, 2007), but we saw no more than a twofold drop in M2 levels (Fig. 3). We were only able to knockdown Aly expression by ∼60 % however (Fig. 2) and although control experiments examining the localization of polyadenylated mRNAs in uninfected siRNA-treated cells showed a degree of nuclear retention (data not shown), it may be that more efficient depletion would reveal a more marked phenotype. Alternatively, our data may indicate a more important role for other cellular adaptor proteins, such as the recently described UIF protein (Hautbergue *et al.*, 2009). A strong requirement for UAP56, another component of the TREX complex and responsible for recruiting Aly to cellular pre-mRNAs, was however seen for export and/or expression of certain viral messages (Fig. 7c, d). UAP56 depletion caused marked retention of segment 7 and 8 transcripts and concomitant reductions in M1, M2 and NS1 expression (Figs 3, 4 and 5). Curiously, however, NS2 expression was resistant to UAP56 depletion, further highlighting that individual viral transcripts show significant differences in their interactions with the cellular export machinery, even when considering the two spliced mRNAs. Also of note was the finding that while UAP56 depletion had little effect on segment 4 localization, (producing less than a twofold increase in the nuclear : cytoplasmic ratio of segment 4 mRNA), a more than threefold reduction in protein expression was seen (Figs 3 and 5). Loss of functional UAP56 has been shown to affect the transport of mRNA within the cytoplasm of *Drosophila* oocytes (Meignin & Davis, 2008), so we speculate that the reduction in HA expression seen here results from an effect downstream of mRNA nuclear export.

Although we have shown that NXF1 and/or UAP56 are required for export of certain viral transcripts, the mechanism(s) by which these factors are recruited to the mRNAs remains to be determined. Maturation of M2 mRNA resembles that of a normal cellular pre-mRNA: intron removal presumably leads to deposition of the exon junction complex, including UAP56, which will then recruit Aly and NXF1 (Fig. 7d). Alternatively or in addition, NXF1 might be directly recruited to the serine/arginine-rich protein splicing factor 2/alternative splicing factor (SF2/ASF) (Huang *et al.*, 2003) as the latter polypeptide has been shown to regulate the extent of segment 7 splicing (Shih & Krug, 1996a). Recruitment of UAP56 and NXF1 to the unspliced transcripts as well as NXF1 to the intronless late genes is less obvious. UAP56 can be directed to unstructured regions of intronless mRNAs, although if this mechanism applied to the influenza virus transcripts more dependence on Aly might have been expected (Taniguchi & Ohno, 2008). Alternatively, the 5′-cap structure of mRNA plays an important role in the export of both spliced and intronless cellular mRNAs, via the cap-binding complex (CBC) recruiting Aly and thus NXF1 (Cheng *et al.*, 2006; Nojima *et al.*, 2007). However, the CBC must be displaced from the 5′-cap of cellular pre-mRNAs by the viral polymerase during the process of cap-snatching and although PB2 releases the mRNA cap early after transcription initiation (Braam *et al.*, 1983), it is not known whether the CBC rebinds to mature viral mRNAs. Arguing against quick re-recruitment of the CBC, splicing of segment 7 mRNA is controlled by the viral polymerase binding to the 5′-end of the mRNA (including the cap structure as well as an internal polymerase-binding sequence) thereby blocking the 5′-most splice donor site (Fig. 7d; Shih *et al.*, 1995). It has also been proposed that the 5′-ends of all viral mRNAs are similarly occupied by the viral polymerase as a mechanism to prevent a futile cycle of utilizing viral mRNAs as cap-donors (Shih & Krug, 1996b), but this proposal has not been tested *in vivo* yet.

Based on numerous precedents from other nuclear-transcribing viruses (Schneider & Wolff, 2009) it is also possible that viral polypeptide(s) act as an adaptor between the viral mRNA and the cellular nuclear export pathway. For instance, it has been suggested that the viral polymerase complex might functionally replace the cellular CBC for the purposes of nuclear export (Shih & Krug, 1996b). It is well established that the viral polymerase interacts with Pol II (Engelhardt *et al.*, 2005; Loucaides *et al.*, 2009; Mayer *et al.*, 2007; Rameix-Welti *et al.*, 2009), potentially placing it in the correct local environment to interact with the export machinery that would normally be recruited co-transcriptionally to a cellular pre-mRNA. Such a mechanism is compatible with the observation that drugs that inhibit Pol II transcription inhibit export of most of the viral mRNAs (Amorim *et al.*, 2007; Vogel *et al.*, 1994; Wang *et al.*, 2008; this study). NP is also a plausible adaptor candidate: non-RNP-associated NP shuttles between nucleus and cytoplasm (Elton *et al.*, 2001; Neumann *et al.*, 1997; Whittaker *et al.*, 1996) as well as interacting with several cellular proteins involved in mRNA biogenesis and trafficking (Josset *et al.*, 2008; Mayer *et al.*, 2007; Momose *et al.*, 2001). While our data here do not support a functionally important role for the NP–hnRNPA1 interaction, they are consistent with (although not proof of) a role for the NP–UAP56 interaction in viral mRNA trafficking. Similarly, circumstantial evidence suggests NS1 might also function as an export adaptor (Schneider & Wolff, 2009). It interacts with NXF1 and other components of the mRNA export pathway (Satterly *et al.*, 2007) as well as with at least a subset of the viral mRNAs (Wang *et al.*, 2008). However, the near normal export of the NP and PB2 mRNAs seen after NXF1 depletion or DRB treatment when NS1 expression is much reduced, as well as a consideration of the kinetics of protein expression in normal cells suggests that if this mechanism does operate at all, it does not do so for the intronless early gene transcripts.

## METHODS

### Materials.

293T and MDCK cells were cultured as described previously (Hutchinson *et al.*, 2008). PR8 virus was grown in cells in serum-free medium in the presence of 1 μg trypsin ml^−1^ and 0.14 % BSA and titrated by plaque assay in MDCK cells as described previously (Hutchinson *et al.*, 2008). Virus infections were performed at an m.o.i. of 10. DRB (Calbiochem) was used at 150 μM and LMB (LC Laboratories) at 10 nM. Rabbit anti-NP, anti-M1, anti-PR8 (HA) and anti-PB2 sera have been described previously (Amorim *et al.*, 2007; Noton *et al.*, 2007). Anti-NS2 serum was the kind gift of Dr Agustin Portela (Agencia Española del Medicamento, 28220 Madrid, Spain). Anti-M2 (clone 14C2), anti-Aly (11G5), anti-UAP56 (ab47955), anti-GAPD (6C5), anti-hnRNPA1 (9H10), anti-NXF1 (53H8) and anti-actin (mAbcam8226) were purchased from Abcam. Secondary antibodies were purchased from Molecular Probes, LiCor Biosciences or DAKO. Plasmids pCDNA-PB2, pCDNA-HA, pCDNA-NP, pCDNA-M1 and pCB8− contain cDNA copies of PR8 segments 1, 4, 5, 7 and 8 (Amorim *et al.*, 2007; Carrasco *et al.*, 2004; Harman *et al.*, 2002; Mullin *et al.*, 2004). siPool siRNAs were purchased from Dharmacon. 293T cells were plated at 0.5×10^5^ cell per well 1 day prior to transfection then transfected with 100 nM siRNA (final concentration) by using Dharmafect-1 following the manufacturer's instructions.

### Microscopy.

FISH was performed essentially as described previously (Amorim *et al.*, 2007). To synthesize mRNA-binding probes, plasmid templates were linearized with *Hin*dIII for pCDNA-M1, *Xba*I for pCB8− and *Kpn*I for pCDNA-PB2, -NP or -HA, and transcribed using SP6 RNA polymerase in the presence of 0.15 mM cyanine 3-UTP (Perkin Elmer) or 25 μM Dig-UTP (Roche). Fluorescence images were captured using a Leica TCS-NT confocal microscope or an Olympus IX70 fluorescence microscope fitted with a Retiga 2000R camera. Post-capture processing was carried out using Adobe Photoshop to apply linear brightness/contrast adjustments evenly across figure panels.

To quantify mRNA nuclear : cytoplasmic ratios, confocal images were analysed using the program ImageJ (NIH). DAPI counterstaining was used to identify the nucleus, which was manually defined using the region of interest (ROI) manager. The cell periphery was also manually delineated using the ROI manager. Nuclear and total cellular intensities for the FISH probe were quantified by using the measure function and the cytoplasmic reading taken as (total-nuclear) intensities. The average intensity of the two compartments was then calculated and used to generate a nuclear : cytoplasmic staining ratio.

### Protein analyses.

Cell lysates were analysed by SDS-PAGE and Western blotting according to standard procedures. Blots were imaged by chemiluminescence by using horseradish-peroxidase-conjugated secondary antibodies and X-ray film, or (for quantification) by fluorescence using IRDye 700/800-conjugated secondary antibodies on a Licor Biosciences Odyssey near-infrared imaging platform. Protein levels were quantified using Licor Odyssey version 3 software using background correction followed by normalization to an actin loading control.
